# Filling the treatment gap: developing a task sharing counselling intervention for perinatal depression in Khayelitsha, South Africa

**DOI:** 10.1186/s12888-016-0873-y

**Published:** 2016-05-26

**Authors:** Memory Nyatsanza, Marguerite Schneider, Thandi Davies, Crick Lund

**Affiliations:** Alan J Flisher Centre for Public Mental Health, Department of Psychiatry and Mental Health, University of Cape Town, 46 Sawkins Road, Rondebosch, Cape Town, South Africa; Centre for Global Mental Health, Institute of Psychiatry, Psychology and Neurosciences, King’s College London, London, UK

**Keywords:** Perinatal depression, Counselling, Task sharing, Intervention development

## Abstract

**Background:**

Perinatal depression is a major public health issue especially in low income settings in South Africa, where there is a shortage of mental health professionals. New psychological interventions delivered by non-specialists are needed to fill the treatment gap. This paper describes the process of developing a manual based task sharing counselling intervention for perinatal depression in Khayelitsha, Cape Town.

**Methods:**

Qualitative semi-structured interviews were conducted with 26 participants, including service providers and service users at a clinic in Khayelitsha in order to explore the feasibility, acceptability and content of a task sharing counselling intervention. The interviews were recorded, translated and transcribed. Themes were identified using the framework analysis approach and were coded and analysed using NVivo v10. After the semi-structured interviews, a workshop was conducted with mental health experts on evidence-based psychological interventions for depression, together with a document review of counselling manuals for community health workers in South Africa.

**Results:**

The findings indicate that a task sharing counselling intervention was acceptable and feasible for depressed women in Khayelitsha, under the following conditions: (1) respondents preferred a female counsellor and felt that clinic based individual sessions should be provided at least once a month by an experienced Xhosa speaking counsellor from the community; and (2) the content of a counselling intervention should include psycho-education on cognitive and behavioural effects of depression, how to cope with interpersonal problems, and financial stressors. Based on these conditions, the review of manuals and expert consultation, key components of the counselling intervention were identified as: psycho-education, problem solving, healthy thinking and behaviour activation. These were included in the final counselling manual.

**Conclusion:**

The development of task sharing counselling interventions for perinatal depression should be informed by the views and needs of local service users and service providers. The study illustrates the manner in which these views can be incorporated for the development of evidence-based psychological interventions, within a task sharing framework in low and middle-income countries.

**Electronic supplementary material:**

The online version of this article (doi:10.1186/s12888-016-0873-y) contains supplementary material, which is available to authorized users.

## Background

Common Mental Disorders (CMDs) are highly prevalent in Low and Middle Income Countries (LAMICs) and are known to cause disability and premature mortality [1–3]. Women in the perinatal period are highly vulnerable to depression and suicide [4, 5] and depressed pregnant women are at risk of experiencing various obstetric and neonatal complications, such as spontaneous pre-term labour and low birth weight [6–8]. Impaired mother-infant relationships and poor uptake of antenatal care together with adverse child development outcomes (including increased frequency of infant diarrhoea and delayed psycho-social development) are also common in depressed mothers, partly due to diminished care-giving capacity [6–8].The perinatal period is defined as the period between conception up to 1 year post-partum [4, 9]. In LAMICs the estimated prevalence rate for Common Perinatal Mental Disorders (CPMD) is one in six pregnant women and one in five postnatal women [10]. Global rates for perinatal depression vary enormously as indicated by a review of 40 countries showing perinatal depression ranges from 0 to 73.5 % [11]. Two systematic reviews in LAMICs show weighted mean prevalence rates of 11.3 and 15.6 % for antenatal depression [10, 12] and 18.3 and 19.8 % for postnatal depression [10, 12]. At a local level, a survey of mothers in Khayelitsha revealed a postnatal depression prevalence rate of 34.7 %, a figure three times higher than samples used as comparisons in high income countries (HIC) [13]. A more recent study, also in Khayelitsha, found 39 % of a sample of 1062 mothers to have depressed mood during pregnancy [7].

A systematic review identified positive associations between common contextual factors in LAMICs such as low education level, chronic unemployment, low household income, poverty, intimate partner violence, rejection of paternity, and HIV/AIDS and CPMDs [7, 10, 12, 14–17]. The factors linked to low socio-economic status and CMDs can lead to a vicious cycle of poverty and poor mental health. This is exacerbated during pregnancy when some women experience increased physical and social demands such as anxiety about childbirth and lack of income or food security for the unborn child [5, 17, 18]. This cycle of poverty and ill-health can also have an intergenerational impact on infants and children of depressed mothers [7, 19, 20].

### The treatment gap and task sharing

Despite depression being treatable, a significant number of depressed women in South Africa live with undetected and untreated depression, and form part of a “treatment gap” which is estimated to be more than 75 % of those requiring a service and not receiving one [21, 22]. LAMICs have the least number of mental health specialists [23], highlighting a need for innovative strategies to reduce the treatment gap. Task sharing (also known as task shifting) is a method of making more efficient use of the available resources by training general health workers, including community health workers (CHWs) to deliver interventions which would normally be rendered by specialists [20, 24]. Task sharing antenatal counselling for depressed women could potentially help improve depressed women’s mood, which in turn can prevent suicide, reduce negative obstetric complications and improve infant outcomes [4]. Task sharing is also a way of providing locally relevant treatment by people from the same community, culture and language thereby making the intervention more culturally appropriate [25, 26]. In this model, CHWs do not replace the specialists, but they can help to support less complex cases in order to reduce the number of people needing specialist care [23, 24]. If a task sharing intervention is provided in the context of a good referral system and quality assurance, this could lead to sustainable narrowing of the treatment gap in vulnerable communities [27–29].

### Evidence of task shared counselling in LAMICs

Task sharing has been shown to be effective in a range of countries; for example, in Chile for depression [30] and in India for people living with schizophrenia [31]. It is crucial for task sharing interventions to be adapted from a pool of evidence-based interventions. There are a number of evidence-based counselling techniques which have been adapted and used in the task sharing context.

#### Cognitive Behavioural Therapy

Cognitive Behavioural Therapy (CBT) suggests that when an individual is depressed or distressed they experience cognitive errors in thinking such as having rigid, distorted judgement about themselves or other people [32]. CBT further suggests that if clients are taught to monitor their negative mood, thoughts and behaviour, they can learn to alter these [32], through techniques like healthy thinking and behaviour activation. CBT has been found to be effective in LAMICs by motivating poverty stricken women to become active and change unhealthy thinking patterns, thereby reducing their depressive symptoms [19]. The Thinking Healthy programme in Pakistan, developed by Atif Rahman and colleagues, demonstrated how lady health workers can be successfully trained to deliver an intervention for depressed women [33].

#### Problem Solving Therapy

Problem Solving Therapy (PST) is brief structured therapy which focuses on interpersonal problems in the present social context through collaborative identification, exploration of problems, and identification and implementation of solutions [34]. Research from Zimbabwe on the Friendship Bench (known as *Chigaro Chekupana Mazano* in Shona) indicates how PST can be used to reduce symptoms of depression and CMDs through providing a safe space for people to come and talk about their problems to a trained CHW counsellor [34].

#### Interpersonal Therapy

Interpersonal Therapy (IPT) is an intervention which focuses on four main interpersonal problem areas, namely grief, role transitions, interpersonal or role disputes and interpersonal deficits that are believed to be at the root of depressive symptoms [35]. Understanding the root of interpersonal stress can help an individual to come up with new ways of responding to their situations as well as reduce the triggers for depressive symptoms. Task shared group IPT has been shown to be feasible and acceptable in South Africa [36] with HIV positive women and been successful in reducing depressive symptoms of men and women in Uganda [37].

### The purpose of this study

The purpose of this study is to describe the development of a task sharing counselling intervention for perinatal depression in Khayelitsha, South Africa as part of the AFrica Focus on Intervention Research for Mental health, South Africa randomised controlled trial (RCT) (AFFIRM-SA)[39–41] In 2000, the UK Medical Research Council (MRC) published a framework for developing, evaluating and implementing complex interventions which was revised in 2008, and again in 2015. The 2008 revised MRC framework proposes a cyclical process with 4 stages including: development, feasibility, evaluation and implementation [38]. The focus of this study is on development and feasibility of the intervention. The final two stages of the intervention development, which look at implementation and evaluation of the intervention will be reported as part of the AFFIRM. (RCT)(AFFIRM-SA)[39–41].

## Methods

### Setting

The study was conducted in Khayelitsha, Cape Town. Khayelitsha is one of the largest townships on the eastern outskirts of Cape Town, nearly 30 kilometres from the city centre, with over 500, 000 residents, the majority of whom are Xhosa-speaking [42, 43]. Townships were developed for Black South Africans through the segregation policies of the apartheid government in (1948–1994). The majority of Xhosa-speaking people living in townships such as Khayelitsha, migrated from the Eastern Cape and live in informal settlements in order to seek employment [42]. High unemployment, violence, crime, substance abuse and intolerable living conditions such as inadequate sanitation and overcrowding are rampant [42–44]. Some of the township’s inhabitants live in corrugated iron shacks without running water and electricity although some live in formal housing [43].

### Study design

The study design used a triangulation of methods by synthesising findings obtained from qualitative formative research, an expert panel discussion and a review of existing counselling manuals, in order to develop the intervention. The semi-structured interviews in the formative research provided information on the feasibility of the intervention and information on the needs and experiences of the participants that should be addressed in the intervention manual. The document review and expert consultations provided information for the structure and content of the manual.

#### Sampling and recruitment

Consecutive sampling was used for service users by recruiting every participant who met the inclusion criteria. Screening and recruitment of service users was voluntary and was conducted at a local antenatal and well-baby clinic Community Health Centre (CHC) in Khayelitsha. After agreeing to participate in the study through signing the consent form, users were screened using the Edinburgh Postnatal Depression Scale (EPDS). The EPDS screens for symptoms of Major Depressive Disorder (MDD) based on answers given by the respondent regarding their symptoms over the past 7 days. The EPDS has been translated and validated in isiXhosa with an optimal cut-off score of 12/13 [45]. Users who scored 13 or more on the EPDS were assessed using the Major depression module of the Mini Neuropsychiatric Interview (MINI) v 6.0 [46]. The women who were diagnosed with a major episode of depression on the MINI were interviewed for this study. Recruited users were asked to give further consent for the interview to be recorded and all user interviews were conducted in isiXhosa by a trained field worker, transcribed and then translated into English. Users who were not recruited were given information on organisations and services available in the area such as social services for additional support.

Sampling of service providers was purposive and voluntary with the aim being to recruit HIV counsellors, midwives and CHWs to explore their knowledge of depression, and ability to deliver the proposed intervention. Selected participants gave written informed consent for participating and for allowing the interview to be recorded. All the service provider interviews were conducted in English by a researcher from AFFIRM.

### Semi-structured interviews

The semi-structured interviews assessed participants’ views on the feasibility, acceptability and content of a task shared counselling intervention for perinatal depression in Khayelitsha. Tables 1 and 2 below provide a list of some of the questions asked in the interviews with service users and service providers. The interview schedules were developed by the AFFIRM team, informed by the relevant literature on psychological interventions for CPMD in LAMICs.

**Table 1:** Service user Interview questions (not the complete interview)

Symptoms of depression 1. Do you think you have depression? Why do you think you have depression? 2. How do these feelings that you have change your daily life? 3. Think about a days when these feelings are really bad. Can you tell me what makes it really bad? Strategies for dealing with depression 4. Can you describe a day you feel better and not so depressed… What makes it better? 5. Was there anything you did yourself? Counselling as an intervention 6. The word/name counselling involves somebody helping you, listening to you talking about your problems, and helping you to find some solution to those problems. It does not mean the counsellor will fix your problems for you, but they can help you find ways to solve some of your own problems by giving you new skills that you can use. This counselling is not the same as HIV counselling. 7. Do you think that counselling could help you with your feelings of depression? 8. In what way could it help you? Logistics of counselling 9. If mental health services are closer to where people live, will it help them to use the services? If you could see a counsellor to help you with depression, would you rather see that person at the clinic or at home? Is distance or transport that make it easy or difficult or money or maybe cost of services or getting someone to go with you? 10. If someone came to your house what will your family think? What will the community think? Characteristics of the counsellor 11. If you could choose, would you want to see a nurse, a community health worker, or an HIV counsellor to get counselling for depression? 12. If you could see a counsellor to help you with depression, would you rather see that person at the clinic or at home? 13. If you could choose, what type of person would you choose to give you counselling? 14. What age should they be? 15. What culture should they be from? 16. Where should they come from? 17. What qualification or training should they have? 18. What language should they speak? 19. How many times in a month would you like to see the person? 20. Would it be best done individually or in a group with other people who are depressed? Please explain. 21. What do you think some of the problems to getting this help might be?

**Table 2:** Service provider interview questions (not the complete interview)

Counselling experience and support 1. Can you explain to me what your understanding of depression is and how you would know if a person has depression? 2. What typical symptoms do people have who are depressed and how do these symptoms affect their lives? How long do these symptoms usually last? 3. How common is depression in Khayelitsha? Are pregnant women and mothers more likely to be depressed than other people? If so, why? 4. What do you think would be the best way to help pregnant women and mothers with depression in Khayelitsha? 5. What kind of support do you think depressed pregnant women and mothers need? 6. Would you say it would be better to have a CHW or HIV counsellor to do the counselling to the mothers? 7. Do you know what mental health counsellors are? What do mental health counsellors do? 8. Do you have any counselling experience? If so, describe this experience. 9. What skills would you like to learn if you were to provide counselling to depressed pregnant women or mothers of young infants in Khayelitsha? 10. What kind of support and supervision would you need in order to do counselling for depressed mothers in their homes? How often would you like to meet with a supervisor/manager? Characteristics of the counsellor 1. Would you say it would be better to have a CHW or HIV counsellor to do the counselling to the mothers? 2. What kind of counsellor is acceptable to depressed mothers or pregnant women? (Gender, age, qualification, culture, race, locality, relationship/community relations). 3. Tell me about your current work - Which organisation you work for? 4. How often do you see your supervisor and how do you report to your supervisor? 5. If you were working with pregnant women and mothers who are depressed, how many home visits do you think you could manage, over a period of 6 months? [*If the person struggles to think about 6 months ask about 1 month or even 1 week and then multiply this by 6 for months and by 26 for weeks]*. 6. When in the day and week do you think such home visits would be possible for you? Logistics of counselling 1. Do you visit patients at their homes? And if yes, how often do you do this? What kind of work do you normally do with patients? 2. How comfortable do you/would you feel visiting patients at home – how acceptable is it? (Probe for issues of confidentiality, safety, e.t.c.) 3. What limitations/obstacles do you see in delivering a counselling programme to depressed patients at their homes? 4. What are the potential benefits of counselling women in their homes or clinics that you wouldn’t get if you counselled them at the clinic?	

### Data analysis

All interview transcripts were uploaded into NVivo v10 and texts were analysed using an *a priori* framework developed from the interview topic guides set out in Tables 1 and 2. The framework approach is a five step process including familiarization, identifying a thematic framework, indexing, charting, mapping and interpretation [47]. Further codes were added to the *a priori* themes based on the emergent themes from the interviews. The first author generated themes and coded the data independently and circulated findings to the rest of the team who then reached consensus on the final coded data after collapsing and reorganising the themes. Of particular interest was the type of feelings experienced by the women and the activities they use to cope with these feelings. These assisted in identifying key activities to be included in the intervention.

### Panel discussion and intervention development

Once the interviews were analysed, a 2-stage panel discussion was held over 1 day. The panel comprised of local South African mental health experts with experience in the field of task sharing, intervention development and delivery of maternal mental health care. In the first stage, the preliminary findings from the semi-structured interviews were presented to the panel. In the second stage, the expert panel discussed the adaptation of evidence-based techniques to fit the local context (as presented in the interview data) for delivery by CHWs. The discussion and recommendations made by the panel were incorporated into the development of the proposed intervention and training manual.

### Manual development

Balaji and colleagues suggest that in the context of task sharing psycho-social interventions it is important to develop a manual as well as a protocol for supervision to promote fidelity and a high quality intervention [31]. The third part of the intervention development was thus the development of the manual through a review of other manuals used to train community health workers in counselling in South Africa and other LAMICs. These manuals included The Perinatal Mental Health Project (PMHP) basic counselling skills guide [48], the Lifeline/Childline basic counselling skills participant manual [49], the STRIVE Booklet by the South African National Council of Alcoholism and Drug Dependence (SANCA) [50], the Thinking Healthy manual [51], the PRogramme for Improving Mental Health carE (PRIME) psychosocial group intervention for maternal depression [52] and a CBT manual for medication adherence and depression (CBT-AD) in HIV-infected patients, a version adapted for South Africa by Safren and colleagues [53]. These were reviewed in light of the interview findings and expert panel recommendations, and subsequently the core components of the AFFIRM perinatal counselling manual were drafted.

### Ethical approval

All study participants gave written informed consent to participate in the study and to have their interviews recorded and findings used for publication. Ethical approval for the AFFIRM SA study was granted through the University of Cape Town Health Sciences Human Research Ethics Committee (HREC Reference no: 226/2011 and 842/2014), the Provincial Department of Health and the local CHC head.

## Results

A number of themes emerged from the analysis of the user and provider interviews, as set out in Table 3.

**Table 3:** Frequency of themes

Theme	Services users	Service providers	Total	Percentage
Counselling as an intervention				
Counselling acceptable	12/12	N/A	12/12	100
Counselling as advice	4/12	N/A	4/12	33
Clinic visits preferred	6/12	3/14	9/26	35
Individual sessions preferred	7/12	N/A	7/12	58
Group sessions preferred	5/12	N/A	5/12	42
Barriers to counselling	4/12	8/14	12/26	46
Middle aged, Xhosa woman,	11/12	5/14	16/26	62
Willingness to counsel & positive attitude	N/A	2/14	2/14	14
CHWs recommended	6/12	8/14	14/26	54
Already had counselling training	N/A	4/14	4/14	29
No skills for counselling	N/A	2/14	2/14	14
Avoidance	9/12	N/A	9/12	34
Aggression and withdrawal	6/12	8/14	14/26	54
Exacerbating factors				
Financial Stressors	5/12	6/14	11/26	42
Anxiety	7/12	5/14	12/26	46
Coping strategies	11/12	N/A	11/12	92

### Phase 1: assessing participant views

#### Samples

The overall sample size for semi-structured interviews was 26 participants including seven depressed pregnant women, five depressed mothers of young children, four CHWs, four HIV counsellors, and six midwives the CHC in Khayelitsha.

#### Counselling as an intervention

Counselling was perceived by all users (12, 100 %) as an acceptable form of intervention for perinatal depression within the community. This was reflected in users reporting benefits from talking about their problems or using forms of informal counselling

*It’s going out from the house to meet a family friend and talk and share our problems and, that’s how I get better. I see that I am a person to other people.* (Pregnant Woman 4)

Counselling also seemed to be associated with advice giving and guidance with problem solving in relation to suicidal ideation. Although all service users felt that counselling was acceptable and feasible, some (4, 33 %) expressed fears about sharing problems which could be a barrier to counselling.

*I can’t go next door to ask for a nappy, if they give me today what about tomorrow. I can’t go there tomorrow. I can’t make my problems hers.* (Pregnant Woman 7)

In addition to the above service users’ reservations, nearly half of all participants, users and providers, (12, 46 %) felt that counselling might not be easily taken up.

*Sometimes it won’t be easy to go for counselling. And also for us it is a cultural thing. Most of the people they don’t value the counselling. What I notice is that people go for counselling when they have a problem. We don’t go when there is no problem… Normally there is this thing of we go when things went very, very bad, it is when you will go for help.* (Community Health Worker 4)

We sought to incorporate the views on counselling into our intervention development, by taking note of the participants’ concerns around expectations in counselling and reservations about using the service once it was available in order to make our intervention locally relevant.

#### Characteristics of preferred counsellor

Although all mothers and pregnant women felt that they could attend counselling sessions at the clinic, (7, 58 %) of all users preferred individual sessions conducted at least once a month, by a middle aged Xhosa speaking female counsellor from the community who had practical experience in terms of “knowing what she was doing” as opposed to education level. Under half of service users (5, 42 %) seemed to prefer group sessions. Midwives had the following recommendations.

*I would say preferably a woman, because I think they will be more comfortable and be able to open up if they are speaking to a woman. Um, with race it, it really doesn’t matter as long as our women understand what she is saying and they can be able to communicate. At least there mustn’t be any communication barrier*. (Midwife 1)

When asked if they could add counselling to their current work, (3, 50 %) of midwives seemed unwilling to take on additional roles and recommended that CHWs take on the task sharing role because they have a more flexible workload and job description. One midwife felt that her workload was already high and also raised an important point of how she does not like to work in psychiatry because of previous family trauma.

*There are always changes within the maternity setting, there are always things that are being added, just an addition of work, but no staff…. I don’t like psychiatry, but I do like psychiatric patients, but I just don’t want to be there! But I do have a background of psychiatry at home, with my aunt, which really affected me in a way that I don’t want to work in psychiatry*. (Midwife 1)

Two CHWs (2, 14 %) expressed feeling helpless when seeing depressed women and felt that they did not have the necessary skills to provide counselling. These CHWs did feel however that they would be able to counsel depressed women if they were provided with adequate training and supervision. All HIV counsellors (4, 100 %) mentioned that they had been trained in basic counselling, but this might not enough to counsel depressed women as indicated below.

*We had some basic training for emotional counselling in our HIV counselling training. If we think someone is depressed, we refer them…* (HIV Counsellor 2)

#### Clinic versus home visits

The study results present conflicting views on location of the proposed counselling, women in this particular community showed a preference for individual clinic based counselling sessions. This would encourage them to leave their homes which in some cases exacerbated their depressive symptoms. A number of participants, both users and providers indicated a preference for clinic based visits (9, 35 %) with reasons such as stigma of being HIV positive, issues of confidentiality and leaving the source of depression; expressed in the following quotes.

*People like to look at other people, they will wonder why the counsellor is at my house.* (Mother 1)

And

*You get away from the family, which is often the problem. It is easier to open up, and you get away from the problem.* (HIV Community Health worker 1)

Although clinic visits seemed to be preferred by most, some providers (3, 21 %) raised important points to consider such as lack of space and long waiting periods at the clinics.

*The mothers would have to wait too long at the clinic. If they get hungry or tired, they sometimes just give up and go home. So we need to be able to refer them straight to a psychologist.* (HIV Community Health Worker 3)

After discussing clinic visit possibilities and challenges, we explored the possibility of doing home visits. This brought up several issues for consideration related to counsellors conducting home based visits.

*Transport will be another obstacle because they are far, some of the mothers stay in shacks, where there are no proper streets, and you know* mos *[colloquial expression], crime is also another thing there. So safety will also be another thing to look at…* (Midwife 2)

### Intervention content

The content of the intervention manual was drawn from the descriptions of users’ symptoms of depression in relation to their context.

A few users (2, 16 %) described a cycle of irritability, aggression, social withdrawal and isolation or aggression and conflict which could exacerbate the depression and cause maladaptive functioning if the individual is increasingly isolated.

*People are scared to talk to me, because they say I am always angry, sitting alone in the room. It affects me. I’m always at home I don’t go anywhere. I’m not in the mood for anything not even for washing, nothing…I don’t like to be with people; when my child does something wrong; even if it’s small I shout at him and make it a big a thing. Even when I regret it, I don’t know how to say sorry.* (Mother 4)

Financial stressors such as lack of income and lack of social support also exacerbated the users’ depression as indicated by nearly half (11, 46 %) of both service users and providers. This was reflected in one mother’s quote.

*When I am short of stuff the baby needs. Not having the money to go buy them and not getting support. Then I have those thoughts if only I did not have it… It’s when the father does not support you. You see yourself alone.* (Mother 2)

Another major exacerbating factor was that of anxiety as reported by nearly half of all participants, both users and providers (12, 46 %), and of particular note was anxiety about HIV testing due to partner infidelity.

*What made me worry was that, when you are pregnant you must have the HIV test. I was worried about that…. What made me worry is that, my husband before was adulterous.* (Mother 3)

### Coping strategies

In order to assess what activities would be acceptable to the users if an intervention was developed, users were questioned on things they do themselves in order to deal with their depression. Most servicer users (11, 92 %) were able to identify their coping strategies as indicated in the following narratives.

*That feeling ends when I listen to music.* (Mother 1)

*If I go jogging at least the brain has some peace of mind. …For now I tell myself that I will never try to kill myself again. These are small things; maybe god has many things in store for me.* (Mother 2)

We sought to incorporate these above examples and other activities into our behaviour activation session of our intervention, thereby building on strategies that local mothers already employ to deal with depression.

### Phase 2: Intervention development

The purpose of the workshop was to use the findings from the formative study together with evidence from other studies on perinatal depression to develop an intervention. We needed to decide on content of the intervention, the number of sessions, location of the intervention and who would deliver the intervention. All the above findings were presented to a panel of mental health experts and outcomes from the workshop are explored below.

#### The task shared intervention

In order to develop the intervention, we looked at recurring themes from the semi-structured interviews, together with feedback from other projects and linked these themes to evidence-based techniques such as psycho-education, problem solving, behaviour activation and healthy thinking. The panel recommended that six to eight individual counselling sessions would be ideal to show an effect since some of the concepts are complex and may require revisiting in a subsequent session. A homework component was seen as important and would also require a number of sessions to ensure adequate discussion on completed homework tasks.

A booklet was recommended as a way of engaging with the participants although concerns were raised about illiterate participants. These concerns were addressed by looking at previous studies in Khayelitsha, which found education levels of 26 % with completed secondary schooling in a sample of 1069 women [7] and 54.1 % primary school education and 45.9 % grade 8 or higher in a sample of 98 women [54]. These findings helped us to anticipate recruiting participants with a primary school level of education. If participants were illiterate, they would be encouraged to discuss alternative options of doing the homework such as discussing their ideas with the counsellors, or asking someone at home to assist them with it thus making the exercises inclusive of other family members. The booklet would be used for homework, scheduling appointments, keeping logs of questions, thoughts and activities. A resource list with local places the participant can go to for help in the community would be included in the booklet.

In order to provide guided self-help in a non-judgemental manner, there has to be rapport, trust and therapeutic elements which can be fostered in consecutive sessions in the task shared intervention [55]. The order of sessions was important and it was necessary to make psycho-education about depression the first session since it would educate the participant on the symptoms of depression and build rapport with the counsellor. The second session on problem solving was included in order to assist participants to address everyday problems such as employment, housing, conflict with partners and HIV diagnosis - common factors associated with perinatal depression [7, 15]. This second session includes steps on how to look for alternative solutions to one’s problems [34, 35].

Behaviour activation was included as the third session to help participants who avoided places that they used to enjoy going to or who slept all day because of their depression. Behaviour activation sessions can assist these participants to come up with plans for activities that they would like to do more frequently as a way of combating their depression [56]. The CHW can encourage participants to continue or take up various activities such as exercise or listening to music when feeling depressed to make them feel better as a suggested strategy by participants in the formative findings. The fourth sessions is about healthy thinking which is in line with CBT and the Thinking Healthy programme developed by Atif Rahman and colleagues in Pakistan [19]. Some of the respondents indicated using healthy thinking as a way of coping with their depression, a strategy which has been shown to be effective in combatting depression [33]. Healthy thinking is especially beneficial to participants who could be feeling ashamed of themselves and stuck in unhealthy patterns of self-blame and suicidal thoughts due to partner infidelity.These feelings were reflected upon by some of the participants in the interviews.

A birth preparation session was included as the fifth session in order to help allay anxiety of dealing with a new baby through educating the participant on bonding with their baby, preparing for labour and what to take with them to the hospital. The sixth and final session focuses on termination and evaluation in order to bring all the sessions together and help the participant to evaluate each session and discuss what helped her the most and what was not helpful.

Table 4 below provides a summary of the content and structure of the intervention.

**Table 4:** Features of the Intervention

Features of the intervention	Description
Theoretical basis	Psycho-education, Cognitive Behavioural Therapy techniques, such as healthy thinking adapted from the Thinking Healthy programme by Atif Rahman, and Behaviour Activation.
Structure of the intervention	Manual based individual therapy and psycho-social support provided over 6–8 clinic based sessions. Session 1: Psycho-education on depression Session 2: Problem solving Session 3: Behaviour Activation Session 4: Healthy thinking Session 5: Psycho-education on birth preparation and Relaxation. Session 6: Termination and evaluation
Structure of the sessions	3 step process in all the sessions Step 1: Introduction – greeting and follow up on issues from previous meeting or session, or homework discussion. Step 2: Exploration – discussion of purpose of the session and topic, probing and clarification of issues Step 3: Termination - homework, follow up date and termination
Tools	Counselling manual, voice recorder, relaxation CD, activity workbook and resource list for participants.

#### AFFIRM counselling manual

The AFFIRM manual was developed in order to facilitate the training of the CHWs and standardise the delivery of the task shared counselling. The manual is divided into four sections. The first section is intended for trainees and covers basic counselling and information about depression. The second section is intended for trainers and includes training activities, such as vignettes and self-reflection exercises for the trainees. The third section is for the counsellors to use post-training and contains a step–by-step guide on how to run the sessions. The fourth section is a participant activity work book for the counsellors’ reference which will be printed separately and given to participants. The manual was translated into Xhosa and formed the basis of a 5-day training for CHWs prior to the start of the pilot phase of the AFFIRM-SA trial. Twelve counsellors from a local NGO were trained using the AFFIRM manual and six were selected for the project. The training was conducted in isi Xhosa by the AFFIRM mental health counsellor (MN) who is a qualified clinical social worker. The training covered assessment of depression and all the six sessions in detail with role plays to enable the CHWs to practice.

#### The delivery of the intervention

The counselling sessions are conducted at the clinic initially to allow participant to speak comfortably depending on the availability of space and the participant’s needs. Subsequently, home visits are conducted when necessary. In addition to the initial 5-day training, on-going weekly supervision, training, debriefing and support is provided for the CHWs throughout the course of the intervention. Supervision is offered weekly on a group basis and bi-weekly on an individual basis, in order to monitor progress during the sessions, detect adverse events, discuss the difficult cases and provide guidance for counselling sessions. The counsellors are encouraged to improve their counselling skills, adhere to the intervention and ethical practice through regular supervision sessions including feedback from fidelity checks. Counselling for the counsellors is also be provided on request by an external organisation to offer emotional support for the counsellors to prevent burnout.

## Discussion

This study set out to describe the development of a task shared counselling intervention for perinatal depression in Khayelitsha. In order to develop the intervention it was necessary to explore whether task sharing counselling for perinatal depression was feasible and acceptable to potential service users and providers. Such participation is believed to be a key ingredient in helping with service user recovery [57]. This formative work is in line with the MRC guidelines for intervention development [38]. The findings confirm through the narratives provided by participants that untreated depression is common in resource scarce communities underscoring the reality of the treatment gap [21, 22]. This is consistent with other findings that highlight staff shortages [23], poverty, low levels of education and other responsibilities which could also prevent women from getting the help that they need.

There were no major differences identified between perceptions of service users and providers on the proposed intervention. We found that task shared counselling for perinatal depression in Khayelitsha was acceptable and feasible provided that certain conditions were met: (1) respondents preferred a female counsellor and felt that clinic based individual sessions should be provided at least once a month by an experienced Xhosa speaking counsellor from the community; (2) the content of a counselling intervention should include psycho-education on cognitive and behavioural effects of depression, how to cope with interpersonal problems and financial stressors. We were able to incorporate these conditions into our intervention development in order to make it locally relevant [25, 26] and to increase the uptake of our intervention.

Clinic based sessions were preferred in order to avoid neighbours thinking participants were too sick to attend the clinic, possibly due to HIV related stigma linked to home based care services for the frail, although there was some acknowledgement that home based services may improve accessibility [23] for example by allowing CHWs to conduct follow up home visits if participants missed clinic appointments. Participants seemed to associate the age and practical experience of the counsellor with a better prospect of advice-giving compared to a younger counsellor who might not have gone through some of the issues they could be experiencing. We therefore had to take all potential barriers and preferences into consideration for the intervention as well as the safety concerns for CHWs doing home visits. A way to overcome these safety issues is by pairing CHWs when conducting home visits and possibly providing them with transport.

Other potential barriers to counselling uptake included difficulty sharing problems with others for fear of being laughed at and fear of burdening other people. Problem perception could also influence uptake of counselling since some individuals could minimise the extent of their problem and refuse the counselling sessions. All the themes brought up in the interviews were taken into account during the intervention development, panel discussion and intervention and training manual development. The wide range of themes called for an eclectic approach [58], which could help women to understand the effect that depression has on their current situation, understand that there are ways of looking at their situations differently, and start taking steps towards changing their situations. Complex elements of the intervention would need to be explained and taught to the counsellors in a clear and understandable manner. The step-by-step guide in the manual that was developed provides the counsellors with instructions on how to conduct the sessions in order to ensure quality and standardised delivery of the intervention [31, 59]. The advantage of an eclectic approach is that it could help the participant to master a range of responses to different situations as opposed to a unitary model of therapy [58]. Some service users also indicated that depression affects their care giving capacity which is consistent with findings from previous epidemiological research on perinatal depression in Khayelitsha [7]. Our intervention aims to teach participants new ways of responding to their situations through PST and CBT techniques such as healthy thinking and behaviour activation, similar to the Friendship Bench intervention in Zimbabwe [34] and the Thinking Healthy programme in Pakistan [19].

For us to identify the best cadre to deliver the intervention, we had to consider motivation for counselling, and it is important to note how previous trauma can affect one’s motivation to work in different fields and how such an individual should be given the option to work in their preferred field which is not always the case when resources are scarce. Although nurses are probably in a good position to deliver the training, their workload is high and they might not be able to incorporate new training into their schedules. Motivation for working in mental health counselling and empathy towards depressed mothers are also important qualities that should be considered when looking at the training and selection of potential service providers. Higher fidelity is likely if those delivering the intervention are adequately trained and supervised and highly motivated to deliver the intervention [59]. Given the interest and motivation displayed by the CHWs interviewed in this formative research, and the agreement by the expert panel that this cadre was best placed to deliver the intervention, we decided to recruit and train CHWs as the counsellors.

### Limitations

We are aware that the sample size was small and could have yielded a limited range of responses, however; the sample size was appropriate as the study was formative and qualitative in nature. We are also aware that the participants may have had limited experience of counselling and therefore may not have been able to identify all the possible elements that might be included in a counselling intervention. The study could have also explored indigenous healing systems that local women use to cope with adversity in order to incorporate these into our intervention development.

## Conclusion

The key elements of the study included qualitative formative research, expert consultation and review of other manuals, all of which proved to be crucial for the development of the intervention. The development and adaptation of task sharing counselling interventions for perinatal depression should be informed by the views and needs of local service users and service providers to determine the acceptable content and form of such an intervention. The study illustrates the manner in which evidence-based psychological interventions can be adapted for use by community health workers within a task sharing framework in low and middle income countries.

## Abbreviations

UDP, uridine diphosphate; β4GalT-I, β-1, 4-galactosyltransferase, α-LA: α-lactalbumin; PKB, the protein kinase B, also known as AKT; GLUT, glucose transporter; qPCR, quantitative real-time PCR; SLC35A2, solute carrier family 35 member A2; SLC35B1, solute carrier family 35 member B1; HK2, hexokinase 2; Scr, scrambled siRNA; FITC, fluorescein isothiocyanate.

## Additional file

Additional file 1: Consolidated criteria for reporting qualitative research (COREQ): 32 Item Checklist. (DOCX 23 kb)
