# Supplementary material for: Filling the treatment gap: developing a task sharing counselling intervention for perinatal depression in Khayelitsha, South Africa
Source: BMC Psychiatry. 2016 May 26;16:164. doi: 10.1186/s12888-016-0873-y (PMC4881204; doi:10.1186/s12888-016-0873-y)
Supplement: Additional file 1: — Consolidated criteria for reporting qualitative research (COREQ): 32 Item Checklist. (DOCX 23 kb) [file 12888_2016_873_MOESM1_ESM.docx]

Consolidated criteria for reporting qualitative research (COREQ): 32 Item Checklist

No Item Guide questions/description

Domain 1: Research team and reflexivity

Personal Characteristics

1. Interviewer/facilitator Which author/s conducted the interview or focus group?

**TD**

1. Credentials What were the researcher’s credentials? E.g. PhD, MD

**Masters in Sociology, Registered Counsellor**

1. Occupation What was their occupation at the time of the study?

**Research Officer**

1. Gender Was the researcher male or female?

**Female**

1. Experience and training What experience or training did the researcher have?

**Previous experience conducting qualitative research from Masters study; training for data collection from the Randomised Controlled Trial.**

Relationship with participants

1. Relationship established Was a relationship established prior to study commencement?

**No. Researcher introduced herself and study to the staff of the community health centre after obtaining permission from the ethics committee and department of health.**

1. Participant knowledge of the interviewer What did the participants know about the researcher? e.g. personal goals, reasons for doing the research

**Participants knew about the goals for conducting the research as part of the informed consent procedure prior to giving consent to participate in the study.**

1. Interviewer characteristics What characteristics were reported about the interviewer/facilitator? e.g. Bias, assumptions, reasons and interests in the research topic

**No bias reported. Possibility of bias eliminated by questions being developed by the team prior to conducting the interviews. The research was assessing the acceptability and feasibility of a task shared intervention for perinatal depression.**

Domain 2: study design

1. Theoretical framework

Methodological orientation and Theory What methodological orientation was stated to underpin the study? e.g. grounded theory, discourse analysis, ethnography, phenomenology, content analysis

**Content analysis (framework approach)**

Participant selection

1. Sampling How were participants selected? e.g. purposive, convenience, consecutive, snowball

**Purposive sampling was used to select participants.**

1. Method of approach How were participants approached? e.g. face-to-face, telephone, mail, email

**Participants were approached face to face.**

1. Sample size How many participants were in the study?

**Twenty six**

1. Non-participation How many people refused to participate or dropped out? Reasons?

**None.**

Setting

1. Setting of data collection Where was the data collected? e.g. home, clinic, workplace

**Data was collected at the community health centre (clinic).**

1. Presence of non-participants Was anyone else present besides the participants and researchers?

**No. Interviews were conducted in private with only the researchers and participants present.**

1. Description of sample What are the important characteristics of the sample? e.g. demographic data, date

**All participants were adult females. Service users and services providers were interviewed: Seven depressed pregnant women, five depressed mothers of young babies, four community health workers, four HIV counsellors and six midwives.**

Data collection

1. Interview guide Were questions, prompts, guides provided by the authors? Was it pilot tested?

**There was a semi-structured interview guide used by the researchers. It was not pilot tested as the data and responses were qualitative.**

1. Repeat interviews Were repeat interviews carried out? If yes, how many?

**No repeat interviews were conducted**.

1. Audio/visual recording Did the research use audio or visual recording to collect the data?

**Audio recording was used to collect the data.**

1. Field notes Were field notes made during and/or after the interview or focus group?

**No field notes were made during the interview.**

1. Duration

What was the duration of the interviews or focus group?

**Interviews were approximately 30 minutes each.**

1. Data saturation

Was data saturation discussed?

**Data saturation was discussed and it was agreed that approximately 26 participants were adequate for representation for the formative qualitative work. In the data analysis, saturation was reached and there was not a need for further interviews.**

1. Transcripts returned Were transcripts returned to participants for comment and/or correction?

**Transcripts were not returned to the participants for comment or correction.**

Domain 3: analysis and findings

Data analysis

1. Number of data coders How many data coders coded the data?

**TD and MN coded the data**

1. Description of the coding tree Did authors provide a description of the coding tree?

**No description of the coding tree. Framework approach to analysis was used instead of coding tree.**

1. Derivation of themes Were themes identified in advance or derived from the data?

**Most themes were derived from the data and some themes were identified apriori as part of the framework of analysis.**

1. Software What software, if applicable, was used to manage the data?

**NVivo v10 software was used to manage the data.**

1. Participant checking Did participants provide feedback on the findings?

**Participants did not provide feedback on the findings.**

Reporting

1. Quotations presented Were participant quotations presented to illustrate the themes / findings? Was each quotation identified? e.g. participant number

**Participant quotations were presented to illustrate the themes/ findings and each quotation is identified by a participant number.**

1. Data and findings consistent Was there consistency between the data presented and the findings?

**There was consistency between the data presented and findings.**

1. Clarity of major themes Were major themes clearly presented in the findings?

**Major themes were clearly presented in the findings.**

1. Clarity of minor themes Is there a description of diverse cases or discussion of minor themes?

**Minor themes were also presented in the findings.**
